# The microsporidian polar tube: origin, structure, composition, function, and application

**DOI:** 10.1186/s13071-023-05908-9

**Published:** 2023-08-30

**Authors:** Yuqing Chen, Qing Lv, Hongjie Liao, Zhengkai Xie, Liuyi Hong, Lei Qi, Guoqing Pan, Mengxian Long, Zeyang Zhou

**Affiliations:** 1 State Key Laboratory of Resource Insects, Southwest University, Chongqing, 400715 China; 2 Chongqing Key Laboratory of Microsporidia Infection and Control, Southwest University, Chongqing, 400715 China; 3 College of Life Sciences, Chongqing Normal University, Chongqing, 400047 China; 4 Biomedical Research Center for Structural Analysis, Shandong University, Jinan, 250012 China

**Keywords:** Microsporidia, Polar tube, Polar filament, Structure, Polar tube proteins, Interaction, Diagnosis

## Abstract

Microsporidia are a class of obligate intracellular parasitic unicellular eukaryotes that infect a variety of hosts, even including humans. Although different species of microsporidia differ in host range and specificity, they all share a similar infection organelle, the polar tube, which is also defined as the polar filament in mature spores. In response to the appropriate environmental stimulation, the spore germinates with the polar filament everted, forming a hollow polar tube, and then the infectious cargo is transported into host cells via the polar tube. Hence, the polar tube plays a key role in microsporidian infection. Here, we review the origin, structure, composition, function, and application of the microsporidian polar tube, focusing on the origin of the polar filament, the structural differences between the polar filament and polar tube, and the characteristics of polar tube proteins. Comparing the three-dimensional structure of PTP6 homologous proteins provides new insight for the screening of additional novel polar tube proteins with low sequence similarity in microsporidia. In addition, the interaction of the polar tube with the spore wall and the host are summarized to better understand the infection mechanism of microsporidia. Due to the specificity of polar tube proteins, they are also used as the target in the diagnosis and prevention of microsporidiosis. With the present findings, we propose a future study on the polar tube of microsporidia.

**Graphical Abstract:**

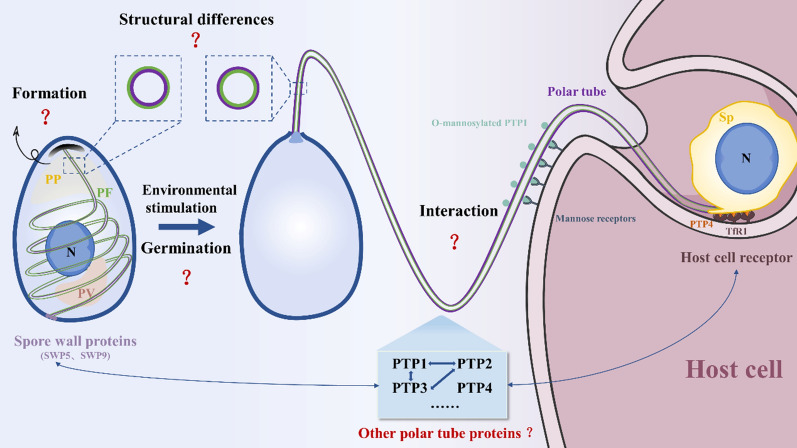

## Background

Microsporidia are a class of obligate intracellular parasitic unicellular eukaryotes. In the mid-nineteenth century, *Nosema bombycis* was first identified as the pathogen causing severe Pébrine disease in silkworms [1]. Initially, microsporidia were classified as protists [2]. With the phylogenetic analysis of the conserved gene (α-tubulin, β-tubulin, RNA polymerase II, heat shock protein 70), data have confirmed that microsporidia are a clade or sister group to fungi [3–13].

Microsporidia have a wide range of hosts, including invertebrates, vertebrates, and even humans [14]. Approximately half of all animal phyla have been reported to be infected with microsporidia [15]. The wide host range enables microsporidia to obtain more survival resources and opportunities [16–18]. More than 200 genera and 1700 species of microsporidia have been identified, 17 of which are known to infect humans, including *Anncaliia connori*, *Anncaliia algerae*, *Anncaliia vesicularum*, *Enterocytozoon bieneusi*, *Encephalitozoon cuniculi*, *Encephalitozoon hellem*, *Encephalitozoon intestinalis*, *Pleistophora* sp., *Pleistophora ronneafiei*, *Trachipleistophora anthropophthera*, *Trachipleistophora hominis*, *Tubulinosema acridophagus*, *Vittaforma corneae*, *Nosema ocularum*, *Endoreticulatus* sp., *Microsporidium africanum*, and *Microsporidium ceylonensis* [19–25]. Although human-infecting microsporidia were generally thought to infect immunocompromised patients, they have now been reported in immunocompetent individuals as well [23, 26–28].

Microsporidia differ in host range and host specificity; however, they all have a unique infection organelle, the polar tube, which is also defined as the polar filament in mature spores. In response to the appropriate environmental stimulation, the spore germinates with the polar filament everted, like a reversed finger of a glove, forming a hollow polar tube [29, 30]. The ejection process of the polar filament is so rapid that *Encephalitozoon* species take less than 500 ms for polar filament firing and passing of the infectious cargo through the polar tube, while the velocity of *A. algerae* emerging in the polar tube is up to 300 µm/s, with only 1.6 s required for the entire process [31]. Finally, the infectious sporoplasm is transported into the host cell to complete the microsporidian invasion [32]. However, the process by which the polar tube mediates the sporoplasm entry into host cells is still unclear. Currently, two phenomena have been observed: one in which the polar tube penetrates the host cell membrane to directly deliver the infectious sporoplasm into the host [33, 34], and the other wherein the polar tube attaches to the host cell membrane, forming invasive synapses to create a protected microenvironment for the sporoplasm, after which the sporoplasm is transported into the host cells by endocytosis [35]. Therefore, the polar tube plays an important role in microsporidian infection.

The polar tube is mainly composed of polar tube proteins (PTPs). With the development of proteomics technology, an increasing number of potential PTPs have been successfully identified [36], which lays a foundation for further research on the infection mechanism of microsporidia. In this paper, we review the origin, structure, composition, function, and application of the microsporidian polar tube in recent years, and provide new insights into the study of this unique infection organelle.

## Origin

In general, the life cycle of microsporidia is divided into three stages: infective phase, proliferative phase, and sporogonic phase [37–39]. Microsporidian development starts with mature spores and ends with mature spores. Mature spore germination initiates the infective phase. One of the most widely accepted hypotheses regarding microsporidian germination is that under a series of stimuli, the water from the environment flows into mature spores to increase the intracellular osmotic pressure, which results in the disordering of the polaroplasts and swelling of the posterior vacuole, pushing the discharged polar filament. There are many factors (such as temperature, ultraviolet radiation, pH, metal ions, digestive enzymes, and so on) that trigger this germination process [40–49]. Next, the infectious sporoplasm transported into host cells begins the proliferative phase, which is the first stage of microsporidian intracellular parasitic life. The initial merogony division produces multiple merozoites, which transform into the sporont by binary fission or multiple fission. Then the spore wall begins to gradually thicken outside the plasma membrane, indicating the entry of the sporogonic stage. In this phase, the sporont undergoes binary fission to form a sporoblast, which finally develops as mature spores. The formation of the extrusion apparatus, involving the polar filament, polaroplast, and posterior vacuole, usually begins during this period. Finally, mature spores are released from the infected host to begin the next new life cycle [37].

Microsporidia do not have the typical Golgi complex (GC), but rather a GC-like structure, which is considered as clusters of vesicles derived from a nuclear envelope and endoplasmic reticulum (ER) in the early proliferate stage [50, 51]. And it was thought to be 300-nm networks of thin branching or varicose tubules in the late sporoblasts and young mature spores of *Paranosema grylli* and *Paranosema locustae* [51, 52]. The polar filament considered to originate from the GC-like structure was first observed in the early sporoblast [30, 52, 53]. Thiamine pyrophosphatase (TPPase), a histochemical marker of the GC, was found on the membranes and the high-electron-dense region of the polar filament in the fish microsporidium *Glugea stephani*, suggesting that the GC-like structure was indeed associated with the polar filament formation [53]. In addition, the ER is thought to participate in polar filament formation. It was found the signal of nucleoside diphosphatase (NDPase), a histochemical marker of ER, was in the core and outer sheath of the polar filament in *G. stephani* [54]. Weidner [55] suggested that the central core of the polar filament was from the GC-like structure vesicles, whereas the outer envelope was derived from the ER. Hence, the end of the tubular vesicle of the ER containing high-electron-dense material may be transported to the GC-like structure to gradually develop the extrusion apparatus [37].

**Fig. 1:**
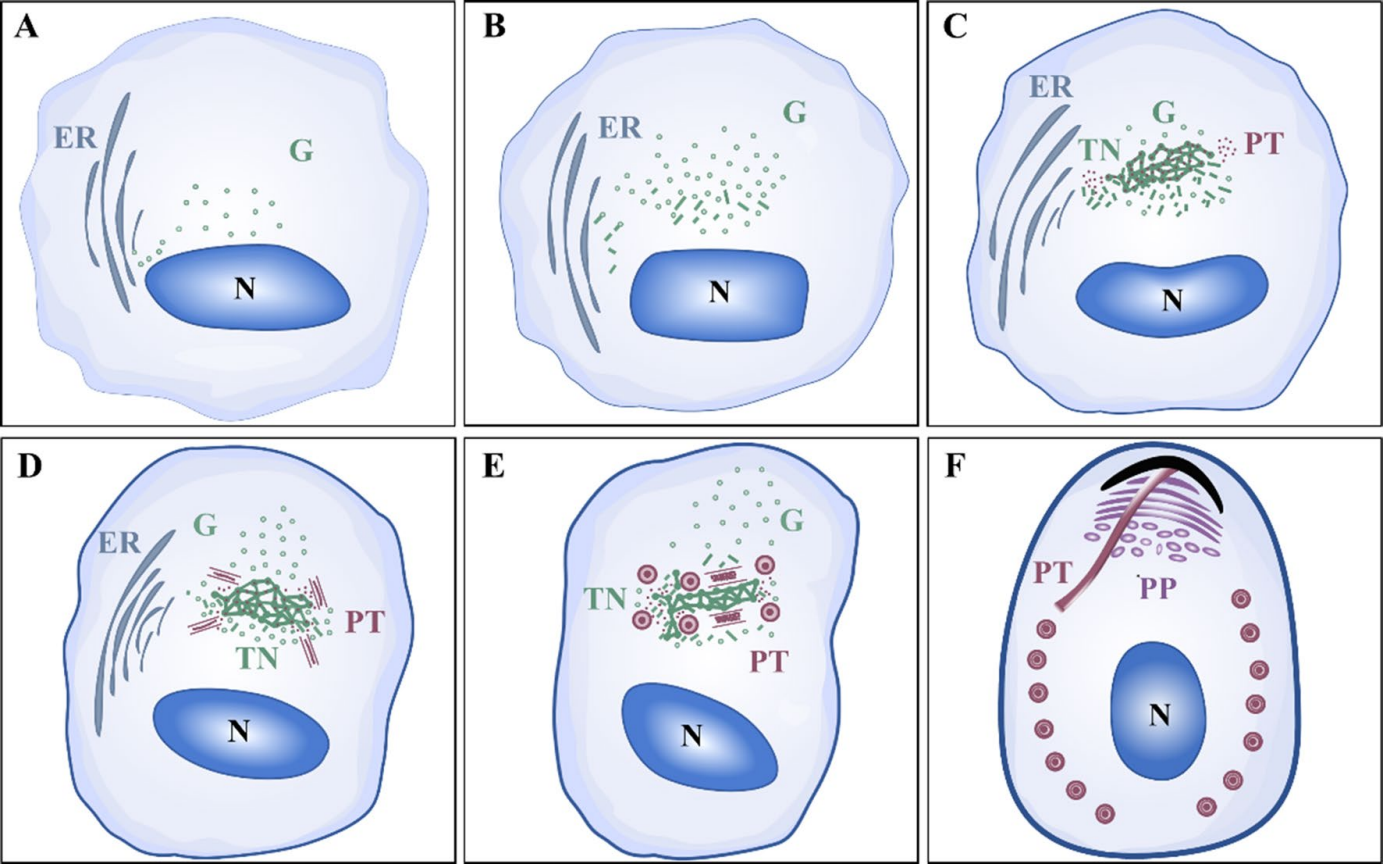
The hypothesis of the formation process of the microsporidian polar filament. **A** In the early proliferate stages, the GC-like structure (G) is considered as clusters of vesicles derived from the nucleus (N) and endoplasmic reticulum (ER). **B** The GC-like structure gradually appears as tubules. **C** With the tubular network (TN) formed, the PTPs are concentrated and modified in the TN and gradually accumulate at its edge. **D** With the assembly of PTPs, the core and envelope of the polar filaments are first formed. **E**, **F** They further develop into layers to develop mature polar filaments

The formation of the polar filament is a relatively complex process, and a hypothesis has been proposed for microsporidian polar filament formation (Fig. 1A–F). Firstly, electron-dense vesicles appear close to the nucleus (Fig. 1A), like the matrix-arranged clusters of small vesicles (CsVm) in *Saccharomyces cerevisiae* as the “ER-to-Golgi intermediates” [51, 56, 57]. Then additional vesicles transform into tightly packed short tubules performing as cis-GC in sporonts (Fig. 1B), similar to tubular clusters of small vesicles (CsVt) in *S. cerevisiae* and vesicular tubular clusters (VTCs) in mammalian cells [51, 56, 57]. With the development of microsporidia, the tubular network (TN) becomes more prominent (Fig. 1C). In addition, the PTPs are synthesized and then are concentrated and modified in the TN [52, 58]. The PTPs gradually accumulate at the edge of the TN to connect with it, forming the core and envelope of the polar filament (Fig. 1D) [52]. Finally, the polar filament is gradually assembled into layers (Fig. 1E, F) [37], depending on its maturity [30, 59].

## Structure

The study of the polar filament structure began in 1960 [49]. The polar filament is composed of two parts: the anterior end is a vertical linear region (the manubrium portion), attached through an anchoring disc and surrounded by the polaroplast, and the other part is the helical region to protect the nucleus at the mid-posterior part of the mature spore [50]. The spiral number of polar filaments usually varies from four to 30 coils, depending on the microsporidian species [30, 31, 50, 60]. The polar filament is a right-handed helix packed at a special angle relative to the anterior–posterior (A–P) axis of the spore, which is 45° ± 10° in *A. algerae* and 37° ± 12° in *Enc. hellem*. This handedness may arise from the composition of polar filaments (such as PTPs) or the mechanical asymmetry involved in polar tube assembly [31]. The polar filament is composed of multiple concentric layers of different electron densities and thicknesses, mainly including the outer dense area, middle electron–lucent area, and inner dense area [29, 30, 49, 61]. Chioralia et al. [62] observed up to six concentric layers in the coiled region and three layers in the manubrium of the *A. algerae* polar filament. Kelley et al. [63] applied the waffle method to reveal the macrostructure of the polar filament in *Enc. hellem*: concentric circles were observed in axial view and cylinders in side view, and 2.5-nm-diameter bumps on the second cylindrical layer of the polar filament. It is widely accepted that the polar filament is located inside the plasma membrane; however, Cali et al. [64] observed the existence of a continuous network of membranes between the polar filament and spore cytoplasm of *A. algerae*, suggesting that the polar tube might be located outside the plasma membrane.

Under suitable stimulation, the polar filament instantly everts from the thinnest end of the spore wall, forming a hollow polar tube. The length of the extruded polar tube is generally 50–500 µm, which is more than twice the length of the polar filament [33, 47]. The diameter of the polar tube is about 0.1–0.2 µm [47, 65–67], and is so elastic that it can be enlarged up to 600 nm to facilitate the traversal of various intracellular cargo [33, 48, 67–69]. The polar tube has the following morphological features: tube-within-tube, cylinder, cylinder-within-cylinder, and unassembled PTPs [33, 46, 64]. Cryo-electron microscopy (cryo–EM) was first used to observe the polar tube of *A. algerae*, demonstrating that the PTPs were regularly arranged to form overlapping sheets, and a multilayer concentric ring structure composed of electron-dense membrane-like materials was found in the polar tube [68]. The surface of the polar tube had a row of ridges at intervals of 5–6 nm, rapidly increasing the diameter of the polar tube to transport the cargo. Some fine fibers were observed on the outermost layer of the polar tubes, which might be the glycosylated PTPs. At the tip of the extruded polar tube of *A. algerae*, a “J”-shaped hook existed [31, 68], which was also found in the extruded polar tube of *N. bombycis* [70]. During the process of germination, the polar tube first elongated to its maximum length , and then rapidly shortened once the sporoplasm was released [31].

## Composition

The key role of the polar tube in microsporidian infection has led to increasing research interest in its composition. A polar tube is composed of proteins, and many polar tube proteins (PTPs) have been reported from microsporidia to date [35, 37, 71–74]. Taking advantage of the special solubility of the polar tube, Keohane et al. [75] treated the microsporidium *Glugea americanus* with 1% sodium dodecyl sulfate (SDS), 9 mol/L urea, and 2% dithiothreitol (DTT), and successively isolated four major proteins with molecular weight of 23, 27, 34, and 43 kDa. The monoclonal antibody specifically recognized the 43-kDa protein localized on the microsporidian polar tube [76–79]. Moreover, this protein contained several cysteine residues at the N-terminal and C-terminal, suggesting that the disulfide bond was important for PTP1 function [72, 75, 80]. Homologous proteins with similar solubility and molecular weight as other microsporidia were also identified and designated as polar tube protein 1 (PTP1) [75, 81, 82]. Moreover, PTP1 is an *O*-mannosylated glycoprotein. Pretreatment of RK13 cells with mannose was found to reduce the infectivity of the microsporidium *Enc. hellem*, implying that *O*-mannosylated PTP1 plays an important role in microsporidian infection [83]. The 35-kDa PTP2 protein was identified by a similar method [82]. PTP2 is more conserved than PTP1 among different microsporidian species, which all have the basic isoelectric point, high lysine content, and conserved cysteine residue sites [82, 84]. Interestingly, the *ptp1* and *ptp2* gene loci of different microsporidia are adjacent, and their neighbor genes are also relatively conserved in *N. bombycis*, *N. ceranae*, *Enc. hellem*, *Enc. intestinalis*, and so on [82, 84, 85]. PTP3 was screened from a complementary DNA (cDNA) library of *Enc. cuniculi*. The molecular weight of PTP3 is approximately 150 kDa. Unlike PTP1 and PTP2, PTP3 only dissolves in the presence of SDS [73]. Moreover, EcPTP1, EcPTP2, and EcPTP3 formed a large protein complex by cross-linkers, and EcPTP3 could interact not only with itself but also with EcPTP1 and EcPTP2 [86]. Hence, PTP3 has been hypothesized to act as a scaffolding protein for polar tube formation [73, 86].

**Fig. 2:**
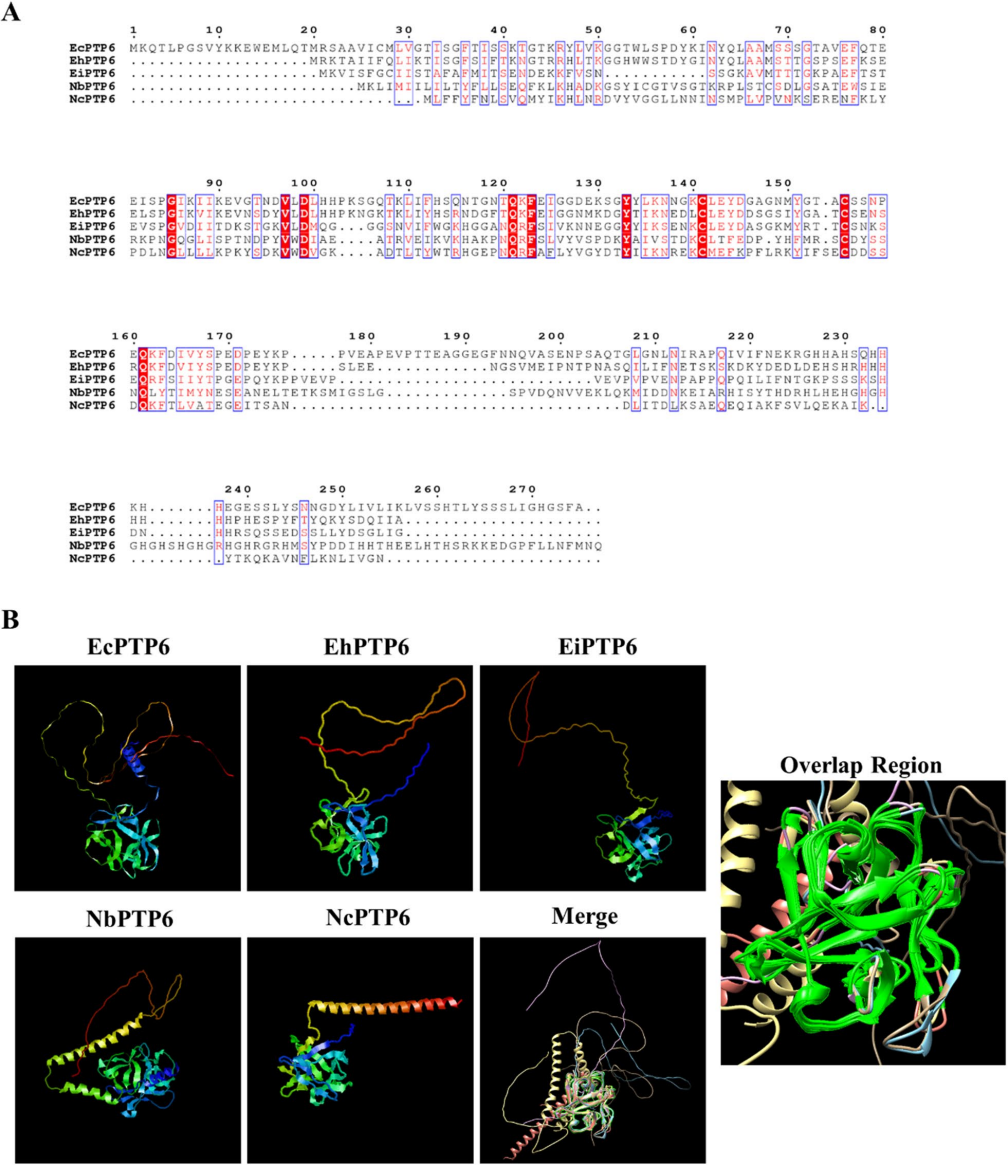
Comparison of the predicted PTP6 protein structure among different species of microsporidia by AlphaFold2. **A** Amino acid sequence alignment was generated with ClustalW (https://www.genome.jp/tools-bin/clustalw) and colored by ESPript 3.0 (https://espript.ibcp.fr/ESPript/cgi-bin/ESPript.cgi). **B** The PTP6 protein structure model of *Enc. cuniculi* (GenBank No. AGE95102.1), *Enc. hellem* (GenBank no. AFM98867.1), *Enc. intestinalis* (GenBank no. ADM12100.1), *N. bombycis* (GenBank no. EOB11485.1), and *N. ceranae* (GenBank no. EEQ82670.1) were predicted by AlphaFold2. The highlighted fluorescent green is the overlap region of PTP6 in different species of microsporidia produced by Chimera 1.16

With the development of proteomics technology, novel PTPs have increasingly been screened and identified. In 2017, EhPTP4 was identified from *Enc. hellem* [35]. Unlike the previous three PTPs, EhPTP4 was specifically localized at the tip of the extruded polar tube and interacted with the special transferrin receptor 1 (TfR1) on the host cell membrane [35]. By comparing the sequence characteristics of PTP1–PTP4 from the genus *Encephalitozoon*, we found that the homologous PTPs have many common features (Table 1). Lv et al. first identified NbPTP6 from *N. bombycis*, which was rich in histidine and serine. Similar to EhPTP4, NbPTP6 could bind with the host cell surface, suggesting that the potential interaction receptor of NbPTP6 is present on the host cell membrane to promote microsporidian infection [74]. Recently, the polar filament and polar tube were isolated and purified from *N. bombycis*. For the analysis of the proteomic composition of these two structures, the candidate PTPs were screened to provide a reference for the identification of novel PTPs [36]. Although the more novel PTPs are screened, the sequence identity of PTPs from different microsporidia is so low that it hinders the finding of homologous proteins by blastp (protein–protein BLAST [Basic Local Alignment Search Tool]) analysis. The development of AlphaFold provides more possibilities for predicting the three-dimensional structure of proteins. Although amino acid sequence identity among the PTP6 homologous proteins from five different microsporidian species is not high (Fig. 2A), they show a highly conservative spatial structure by AlphaFold2 [87, 88]. The overlap region is concentrated at the 10th to 185th amino acid region of PTP6, whose secondary structure is mainly beta-strand and a small amount of random coil (Fig. 2B). In the future, combining the multiple sequence alignment with the protein structural alignment is a recommended method of exploring more novel homologous PTPs with low sequence similarity among different microsporidian species. However, no PTP structure has been resolved until now. Obtaining more information on the structural characteristics of PTPs provides insights into the evolution and function of these proteins, and also for revealing the PTP assembly on the microsporidian polar tube.

**Table 1:** Comparison of the sequence characteristics of PTP1–PTP4 from the genus *Encephalitozoon*

Protein	Gene symbol	Number of amino acids	*pI*	Major amino acid	Number of cysteine	Signal peptide	*O*-glycosylation site	*N*-glycosylation site	Subcellular location
EcPTP1	ECU06_0250	395	4.5	P	17	NF	Y	Y	On the whole PT of *Enc. cuniculi* [71]
EiPTP1	Eint_060150	371	4.3	P	17	Y	Y	NF	
EhPTP1	EHEL_060170	413	4.2	P	19	Y	Y	Y	
ErPTP1	EROM_060160	380	4.4	S	15	NF	Y	Y	
EcPTP2	ECU06_0240	277	8.6	K	8	Y	Y	NF	On the whole PT of *Enc. cuniculi* [82]
EiPTP2	Eint_060140	275	8.6	K	8	Y	Y	Y	
EhPTP2	EHEL_060160	272	8.8	K	8	Y	Y	Y	
ErPTP2	EROM_060150	274	8.8	K	8	Y	Y	Y	
EcPTP3	ECU11_1440	1256	6.1	A	1	Y	Y	Y	On the whole PT of *Enc. cuniculi* [73]
EiPTP3	Eint_111330	1256	5.3	E	1	Y	Y	Y	
EhPTP3	EHEL_111330	1284	6.1	A	1	Y	Y	Y	
ErPTP3	EROM_111330	1254	6.5	A	1	Y	Y	Y	
EcPTP4	ECU07_1090	276	7.1	E	6	Y	Y	Y	On the tip of the PT of *Enc. hellem* [35]
EiPTP4	Eint_071050	279	7.3	E	6	NF	Y	Y	
EhPTP4	EHEL_071080	278	7.9	E	6	Y	Y	Y	
ErPTP4	EROM_071050	280	8.4	E	6	NF	Y	Y	

“Ec” = *Encephalitozoon cuniculi*, “Ei” = *Enc. intestinalis*, “Eh” = *Enc. hellem*, “Er” = *Enc. romalae*, PT = polar tube

“Y” = YES, “NF” = not found

The signal peptide was predicted by SignalP 4.1 (https://services.healthtech.dtu.dk/services/SignalP-4.1/)

NETNGLYC (https://services.healthtech.dtu.dk/services/NetNGlyc-1.0/) and NETOGLYC (https://services.healthtech.dtu.dk/services/NetOGlyc-4.0/) were used to analyze *N*- and *O*-glycosylation sites

## Function

The polar tube acts as a bridge for transporting the infectious cargo into host cells, which is the key function of the polar tube [30, 33, 36, 37, 58, 89, 90]. Live-cell imaging of nuclei transport through the *A. algerae* polar tube showed that the nuclei were elongated, then recovered a globular shape after exiting the polar tube [31]. Lv et al. labeled the diplokaryons of *N. bombycis* and also found that they were elongated for transport in the polar tube [70]. However, Takvorian et al. [68] observed some circular diplokaryons surrounded by a membrane forming the oval or sperm-head-shaped structures in the *A. algerae* polar tube. Therefore, understanding how the nuclei are transported through the polar tube still requires further evidence. The anterior end of the extruded polar tube would form a “J”-shaped hook [68], where the droplet sporoplasm attached to the polar tube for several minutes [33]. In 2019, the interaction between sporoplasm surface protein 1 (EhSSP1) and EhPTP4 was found, contributing to sporoplasm adhesion at the tip of the polar tube [91].

Polar tubes may also interact with host surface proteins. Han et al. [35] analyzed the interaction between EhPTP4 and the host TfR1, which was the first identified host receptor for PTPs. TfR1 recombinant protein and anti-TfR1 antibody had an obvious inhibitory effect on the microsporidian infection rate in TfR1 knockout cells [35]. Moreover, it was also found that mannose-pretreated RK13 cells reduced *Enc. hellem* infection, implying that *O*-mannosylated PTP1 might play an important role in interacting with mannose receptors on host cells to promote microsporidian infection [83].

As the outermost structure of mature spores, the spore wall plays an important role in maintaining homeostasis and fixing the polar filament in mature spores. At present, studies on the interaction between the spore wall and polar tube are mainly in the microsporidium *N. bombycis*. As spore wall proteins, NbSWP5 and NbSWP9 were also reported to be localized on the extruded polar tube [92, 93], and NbSWP5 was found to interact with NbPTP2 and NbPTP3 [93]. Meanwhile, NbSWP9 interacted with NbPTP1, NbPTP2, and NbPTP3, which was considered to be a scaffolding protein to anchor the polar tube fixed to the spore wall for protection of the nucleus in mature spores [92].

## Application

As a pathogen with such a broad infection range, microsporidia not only threaten human health, but also cause serious economic losses for the aquaculture industry [94]. Therefore, the diagnosis and control of microsporidia have become an important research target in recent years (Table 2).

**Table 2:** PTPs as the target application in the diagnosis and control of microsporidia

Applications	Target	Species	Host	Methods	References
Diagnosis	*ptp2* (MT249228.1)	*Enterocytozoon hepatopenaei*	Shrimp	RPA and CRISPR–Cas12a	[98]
	*ptp2* (MT249228.1)	*Enterocytozoon hepatopenaei*	Shrimp	SYBR Green I fluorescence qPCR	[99]
	*ptp3* (XM_002996713.1)	*Nosema ceranae*	Honey bee	LAMP	[100]
	*ptp1* (AJ005666)	*Encephalitozoon cuniculi*	Human	PCR	[78]
Control	*ptp3* (XM_002996713.1)	*Nosema ceranae*	Honey bee	Oral administration of dsRNA	[103]
	PTP1 (XP_003072984.1)	*Encephalitozoon intestinalis*	Human	Treat with anti-polar tube sera	[104]

CRISPR, clustered regularly interspaced short palindromic repeats; dsRNA, double-stranded RNA; qPCR, quantitative polymerase chain reaction; RPA, recombinase polymerase amplification

The microscopic examination method for *N. bombycis* invented by Pasteur is still used in China today, and is a low-cost and simple operation; however, it has great limitations in sensitivity and specificity [95–97]. Therefore, PTPs are unique and conserved to utilize as the microsporidian diagnostic target in modern molecular detection methods. With *ptp2* as the detection target, Kanitchinda et al. (2020) used recombinase polymerase amplification (RPA) and clustered regularly interspaced short palindromic repeats (CRISPR)–Cas12a fluorescence methods to detect *Ent. hepatopenaei* in the hepatopancreas tissue of shrimp [98]. Similarly, a SYBR Green I fluorescence quantitative PCR method targeting the *ptp2* gene was established which, combined with Fluorescent Brightener 28 staining, could be applied to detect and analyze the quantity of *Ent. hepatopenaei* in the field shrimp [99]. Lannutti et al. [100] developed a loop-mediated isothermal amplification (LAMP) assay targeting the *ptp3* gene for rapid detection and monitoring of *N. ceranae* in honey bees. Due to the length polymorphism and sequence diversity, *ptp1* genes have gradually become a good candidate target for analysis of the species type of human-infecting microsporidia (such as *Ent. bieneusi* and *Enc. cuniculi*), providing a supplementary method for genotyping [77, 78]. According to a large-scale serosurvey reported, an immune response to the polar tube of *Enc. intestinalis* appeared in 8% of Dutch blood donors and 5% of pregnant women in France [101]. In this case, the serological diagnostic method of targeting PTPs (based on immunofluorescence antibody staining, enzyme-linked immunoassay [ELISA], and western blotting) is an important tool for studying the pathogenicity and epidemiology of human-infecting microsporidia.

In light of the limited drugs available for treatment (only albendazole and fumagillin are currently approved for microsporidiosis) [102], PTPs have potential importance in the control of microsporidia. Rodriguez Garcia et al. [103] silenced the *ptp3* gene expression of *N. ceranae* in bees by oral administration of *ptp3* double-stranded RNA (dsRNA) to achieve microsporidian load reduction, which provided a novel idea for the prevention and control of microsporidia in RNA interference (RNAi) therapy. In addition, human anti-polar tube sera could partially reduce the microsporidium *Enc. intestinalis* infection in vitro, which was also thought to be a potential microsporidiosis treatment [104].

## Conclusion

**Fig. 3:**
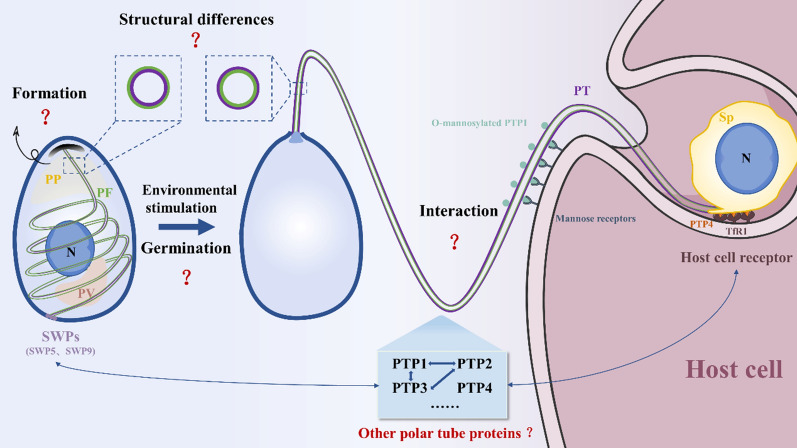
Summary. In response to external environmental stimulation, the spore germinates with the polar filament everted, forming a hollow polar tube, and then the infectious cargo is transported to inject into host cells through the polar tube. During this process, the polar tube interacts with both the microsporidian spore wall proteins and host receptors, and there are still many unknown aspects that need to be solved. *PT* polar tube, *PF* polar filament, *PP* polaroplast, *PV* posterior vacuole, *Sp* sporoplasm, *TfR1* transferrin receptor 1

For one and a half centuries, as the special and unique infection organelle, the polar tube has been the research focus of microsporidia. With the continuous development of proteomics and bioinformatics technology, an increasing number of potential PTPs will be screened, laying the foundation for analyzing the composition of the polar tube. In addition, with the help of cryo-EM technology, the structural characteristics of the polar tube and polar filament are gradually becoming clear. However, many aspects of the microsporidian polar tube are not yet understood. In the future, the study and application of the polar tube can be carried out from the following perspectives (Fig. 3): (1) The polar filament is considered to form in the early sporoblast, but the key factor for activation of the polar filament formation is unclear. (2) To clarify the process of the polar filament transformation into the polar tube, the characteristics of PTP assembly on the polar filament need to be demonstrated. (3) The study of the interaction between the polar tube and host is needed in order to reveal the infection mechanism of microsporidia. (4) Finally, additional novel PTPs can be applied for the diagnosis and control of microsporidia.

## Data Availability

No data were collected for this review. All data and information synthesized in the review are already published and publicly available, and those publications are properly cited in the submission.

## Abbreviations

PTPs: Polar tube proteins
GC: Golgi complex
Cryo-EM: Cryo-electron microscopy
TfR1: Transferrin receptor 1
